# Value, development challenges, and strategies for gaining internal endorsement of digitally connected subcutaneous drug delivery devices: a survey of pharmaceutical stakeholders

**DOI:** 10.3389/fdgth.2026.1684685

**Published:** 2026-03-26

**Authors:** Aaron Swick, S. Prasad Peri, Tanisha Hill, Mohamed Datoo, David Morra, Mehul Desai, Omar Rahman

**Affiliations:** 1 Enable Injections, Inc., Cincinnati, OH, United States; 2 Eli Lilly and Company, Indianapolis, IN, United States; 3 Teva Pharmaceutical Industries Ltd., Tel Aviv, Israel; 4 Johnson & Johnson, New Brunswick, NJ, United States; 5 Merck & Co., Inc., Rahway, NJ, United States

**Keywords:** connected health, connected healthcare, connectivity, digital health, digital medicine, drug-device combination product

## Abstract

**Introduction:**

Connected drug delivery devices such as combination products that integrate traditional drug delivery systems with digital connectivity features represent an opportunity to improve treatment outcomes and disease management. This online survey study was conducted to explore the evolving landscape of digitally connected subcutaneous (SC) drug delivery devices, including the perspectives of pharmaceutical stakeholders regarding the promise of these technologies, particularly in relation to the expansion of traditional mobile companion applications and their integration in drug delivery systems.

**Methods:**

A total of 80 employees of pharmaceutical, biotechnology, or digital health companies with primary roles in medical affairs, commercial, combination product development, or digital health who had experience working on SC drug-device combination products completed the survey. Survey questions explored the value propositions of connected SC drug delivery devices for patients, providers, and payers; barriers to the adoption of these technologies; and strategies for gaining internal support for connected healthcare initiatives.

**Results:**

Responses demonstrated that industry professionals recognize the potential value of connected SC drug delivery devices and associated companion mobile applications and are investing in bringing them to market. Nearly all respondents (97.5%) reported that connectivity is at least moderately important to achieving important objectives, including acquiring real-world data, improving medication adherence, and enhancing ease-of-use for patients. Equal potential value was noted for using connectivity in clinical trials or commercial settings, with neither considered more beneficial than the other. Indications in oncology and endocrinology were considered to be the most likely to benefit from connected SC drug delivery devices. Key barriers to the adoption of connected SC drug delivery devices were development cost, data security, and patient and payer acceptance, while generating evidence of internal and external value was noted as a significant barrier to gaining company endorsement.

**Discussion:**

These results should guide strategies for the effective integration of connected healthcare solutions within the pharmaceutical sector.

## 1 Introduction

Noncommunicable chronic diseases account for seven of the ten leading causes of death globally (1). In the United States, 60% of adults have at least one chronic disease, and 40% have two or more (2); in combination with longer lifespans (3) and a corresponding increase in the prevalence of chronic diseases, this has resulted in significant burdens on the healthcare system. Medication is a cost-effective treatment approach. However, nonadherence to treatment for patients with chronic conditions, reported in 7%–93.1% of patients depending on the condition (4–12), limits therapeutic effectiveness and increases healthcare costs. The cost of medication nonadherence is estimated at $100–290 billion in the United States and €1.25 billion in Europe (13).

Nonadherence can occur for a multiplicity of reasons, including mistrust in healthcare providers or systems, medication cost, inadequate provider-patient communication, lack of patient understanding or satisfaction, patient beliefs and preferences, needle phobia, low numeracy or health literacy, fear of side effects, forgetfulness, conflicting medical information, depression, younger or older age, uncoordinated care, polypharmacy, patient-provider race non-concordance, cost and access, hospital discharge, and insufficient social support (4, 12, 14, 15). Adherence measurement methods such as pill counts, self-report measures, and pharmacy claims remain imperfect, with self-reporting in particular consistently overestimating adherence (4, 16). For example, one imperfect measure is PDC (Proportion of Days Covered) rates which measure oral medication adherence by calculating the percentage of days a patient has access to their drug, with 80% or higher typically considered adherent. Improving adherence therefore requires multifaceted approaches that may include lower costs and improved access, changes in clinical workflows, patient engagement, and more effective use of technology, such as electronic health records, mobile health applications, text messaging, and connected drug delivery devices (4, 17).

Connected drug delivery devices integrate traditional drug delivery systems with digital connectivity to capture objective data on medication administration (18–20). These devices can automate dose logging, provide reminders, and transmit real-time usage data through companion software applications (20). Connected drug delivery devices such as combination products that integrate traditional drug delivery systems with digital connectivity features represent a significant opportunity to improve treatment outcomes and disease management for a variety of neurological, hematological, endocrinological, respiratory, autoimmune, and other chronic diseases. These devices collect and transmit usage data while providing features like dosing reminders and adherence tracking through companion software applications. Unlike standalone health applications, connected drug delivery devices directly integrate with the therapeutic product to capture objective data about medication usage and administration for clinicians, researchers, and patients (21).

Connected autoinjectors like RebiSmart (Merck) and BETACONNECT (Bayer) support subcutaneous (SC) injection of biologics (22, 23). These devices can improve ease-of-use and support adherence to treatment regimens (24). Healthcare providers can benefit from objective adherence data, enhanced disease monitoring, and improved treatment decision-making (25). Additionally, payers gain access to real-world evidence to support value-based care models, in which health outcomes are measured against the costs of associated healthcare services and treatments (20, 26). While these devices have several potential benefits, evidence of their impact on treatment adherence and health outcomes remains largely limited to uncontrolled cohort studies (20). However, to date, RebiSmart has shown adherence of 92.6% across 36 months (27), while SmartInhaler has been shown to improve adherence from 30% to 84% across six months. SmartInhaler also reduced exacerbations at two months (6% vs. 24%) and showed improvement in asthma morbidity scores across six months in the same randomized trial. The Propeller system reduced hospitalization days from 2.7 to 0.6 (79%), inpatient days from 7.9 to 1.4 (82%), and emergency department visit days from 19.2 to 8.3 (57%); moreover, it reduced rescue inhaler use by 75%, asthma-free days by 39%, and increased the proportion of patients with controlled asthma compared with routine care (63% vs. 49%).

In addition to a shortage of high-quality data demonstrating the impact of these devices on treatment adherence and health outcomes, there is little published information about how pharmaceutical companies, the primary stakeholders of these technologies, view their potential value and development challenges (28). Several recent high-profile product discontinuations [e.g., t:connect (29), Abilify MyCite (30), Digihaler (31), and Zecuity Patch (32)] have likely influenced industry perceptions of risk, sustainability, and commercial viability and may be causing a lack of trust or commitment to connected components. Improving our understanding of how the industry perceives connected health technologies should therefore help organizations gain confidence in moving forward with their development and accelerate their adoption in clinical practice, particularly during a period of reassessment across the digital health sector.

This study was therefore designed to examine the perspectives of pharmaceutical and digital health stakeholders with direct experience developing connected SC drug delivery devices. The specific objectives were to:

1. Define value propositions of greatest interest to the pharmaceutical, biotechnology, and digital health industries;
2. Identify internal challenges to development within these organizations;
3. Characterize external barriers to adoption by patients, providers, and payers; and
4. Determine effective strategies for addressing these challenges and barriers.

## 2 Methods

### 2.1 Participants

Participants in this study were individuals with relevant experience working at pharmaceutical, biotechnology, or digital health companies. Inclusion criteria included the following: 1) current employees of pharmaceutical, biotechnology, or digital health companies with primary roles in medical affairs, commercial, combination product development, or digital health and 2) experience working on SC drug-device combination products (e.g., autoinjector, syringe, on-body injector/delivery system); 3) experience contributing to projects for which the use of a SC drug delivery device with digital connectivity functionality (defined as acquiring digital information about device use or injections from the delivery device) was considered; and/or 4) experience with SC combination products for which the use of a patient companion mobile application was considered. Participants were excluded if they did not meet the inclusion criteria or if their responses did not meet the third-party survey vendor's quality assurance standards (i.e., they answered questions too quickly, provided low-quality open-text responses, or selected the same or nearly the same answer for all questions).

### 2.2 Survey

This online survey study was conducted via email between March 21 and April 5, 2024, by an independent third-party vendor with a database of over 1.5 million respondents globally. The survey included Likert scale, free-text, and multiple choice questions about topics related to connected SC drug delivery combination products and the integration of connectivity features into these devices, including potential value for patients, prescribers, and payers; the current position of pharmaceutical/biotechnology companies in pursuing the development of connected SC drug delivery devices for use in clinical trials and commercial settings; internal and external barriers to adoption, and strategies for gaining internal support within companies.

### 2.3 Statistical analysis

Complete responses to survey questions were tabulated, and basic descriptive statistics were calculated from survey data. Where Likert scales were used, response options are presented as number and percentage of participants [*n* (%)] or as a weighted mean [standard deviation (SD)]. In questions with binary responses, the number and percentage of participants [*n* (%)] are presented. Free-text responses were manually coded, and codes were analyzed for frequency in order to identify common themes across responses. No formal hypothesis testing was performed, and no sample size was calculated, but a sample of 20 participants each in combination product development, commercial, digital health, and medical affairs for a total of 80 participants was prespecified.

### 2.4 Ethics

For informed consent, at the outset of the survey, participants were required to agree to the survey vendor's compliance statement. This compliance statement included stipulations that participants would be compensated for their time, that they were legally allowed to disclose information asked about in the survey, and that they had read the vendor's privacy policy. Respondents who chose not to agree to the compliance statement were unable to complete the survey and were excluded from the sample.

## 3 Results

### 3.1 Participant professional experience and geographic location

**Figure 1:**
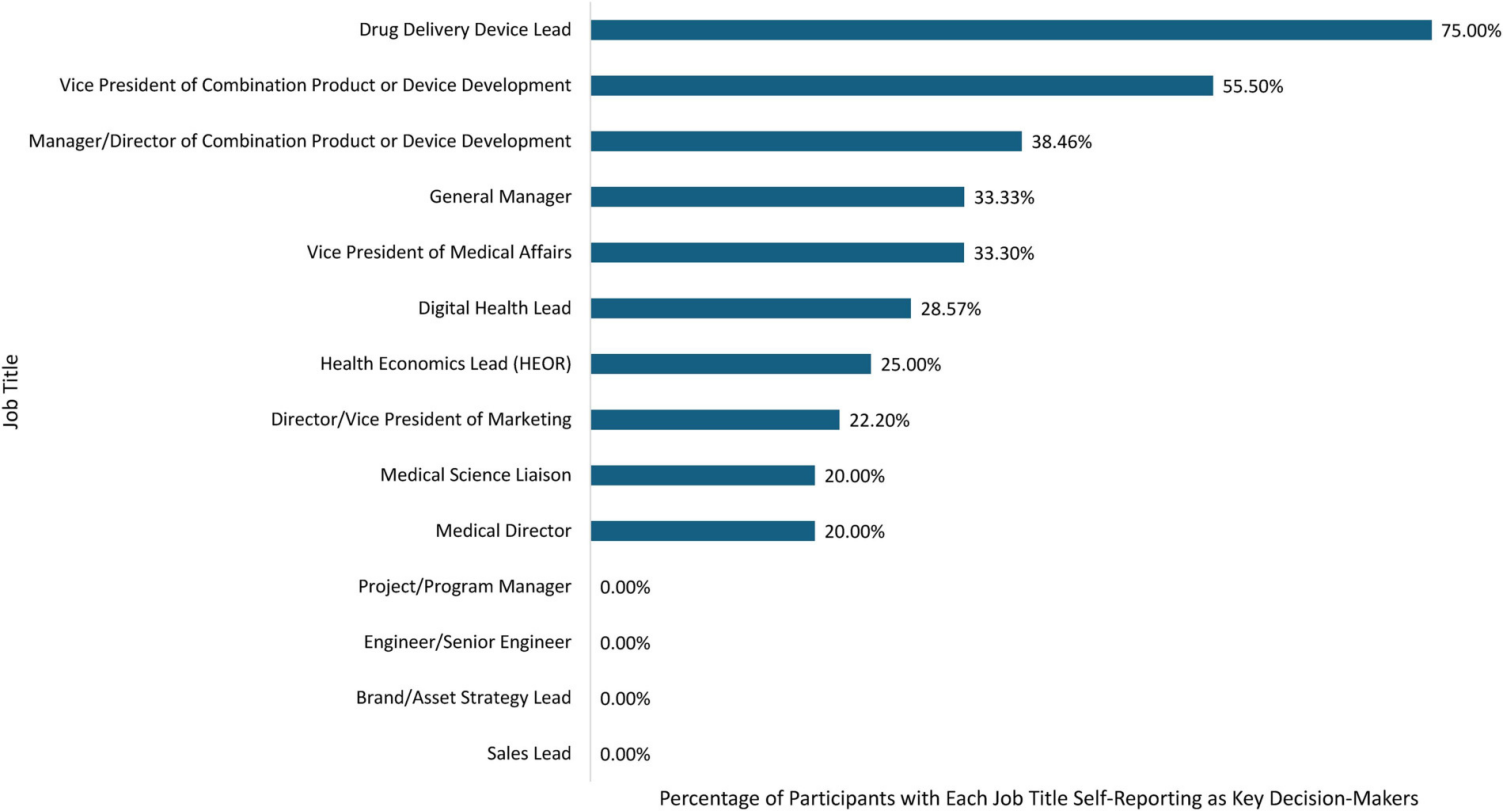
Percentage of respondents per job title who self-reported as key decision-makers.

Nineteen responses were excluded for low-quality data, and 687 were excluded according to the exclusion criteria. A total of 80 eligible participants were included in the study; the characteristics of the sample are summarized in Table 1. Nearly two-thirds (65%) were working at large-cap or mid-cap companies and over half (56%) held positions as Directors or Vice Presidents. Respondents specialized in medical affairs (25%), commercial (25%), combination product development (25%), and digital health (25%); participants had a weighted mean of 10.1 years of relevant experience. All participants had some level of experience working on SC drug-device combination products, with 75% having direct experience contributing to these projects. When asked to indicate main therapeutic areas of experience, oncology was the most represented area, selected by 51 participants (63.75%), followed by endocrinology (41.25%), neurology (33.75%), rheumatology (32.50%), gastroenterology (25.00%), dermatology (23.75%), primary care (22.50%), benign hematology (10.00%), nephrology (8.75%), and other therapeutic areas (3.75%) (participants were able to select more than one therapeutic area). Most respondents (79%) were based in the US; the remainder were based in Belgium, Canada, Germany, Ireland, Japan, Korea, Netherlands, Portugal, Spain, Switzerland, and the United Kingdom. Most (64%) were involved in the decision-making process for SC drug-device combination products, with 30% reporting being the primary decision-maker (Figure 1).

**Table 1:** Participant professional experience and geographic location.

Characteristic	Total sample (*n* = 80)
Specialization^1^	*n* (%)
Combination product development	20 (25.00)
Commercial	20 (25.00)
Digital health	20 (25.00)
Medical affairs	20 (25.00)
Company Type^2^	*n* (%)
Large-cap pharmaceutical company (>$10 billion market capitalization)	31 (38.75)
Mid-cap pharmaceutical company ($2–$10 billion market capitalization)	21 (26.25)
Small-cap pharmaceutical company ($250 million-$2 billion market capitalization)	13 (16.25)
Micro-cap pharmaceutical company (<$250 million market capitalization)	8 (10.00)
Digital health solution developer	7 (8.75)
Experience type^3^	*n* (%)
Both clinical trial and commercial use	38 (47.50)
Exploring concepts, but have not yet used	22 (27.50)
Clinical trial use only	12 (15.00)
Commercial use only	7 (8.75)
Not developing connected drug delivery devices	1 (1.25)
Title/Role^4^	*n* (%)
Vice President/Director/Manager	45 (56.25)
Project or Program Manager/General Manager/Lead	28 (35.00)
Other (Engineer or Field Medical)	7 (8.75)
Therapeutic area(s)^5^	*n* (%)
Oncology	51 (63.75)
Endocrinology	33 (41.25)
Neurology	27 (33.75)
Rheumatology	26 (32.50)
Gastroenterology	20 (25.00)
Dermatology	19 (23.75)
Primary care	18 (22.50)
Benign hematology	8 (10.00)
Nephrology	7 (8.75)
Other	3 (3.75)
Country^6^	*n* (%)
US	63 (78.75)
Other	17 (21.25)

All data shown as *n* (%).

^1^Specialization: “What best describes your primary area of specialization?”

^2^Company Type: “Which of the following best describes your organization?”

^3^Connected Device Experience: “Which of the following statements best describes your company's experience with bringing connected drug delivery devices to market?”

^4^Title/Role: “Which of the following best describes your title/role?”

^5^Experience: Therapeutic Areas:“What are the main therapeutic areas of your experience with subcutaneous drug products? Please select all that apply”

^6^Country: “In which region do you currently work / reside?”

### 3.2 Perceived value of connected SC drug delivery devices for clinical trials or commercial settings

**Figure 2:**
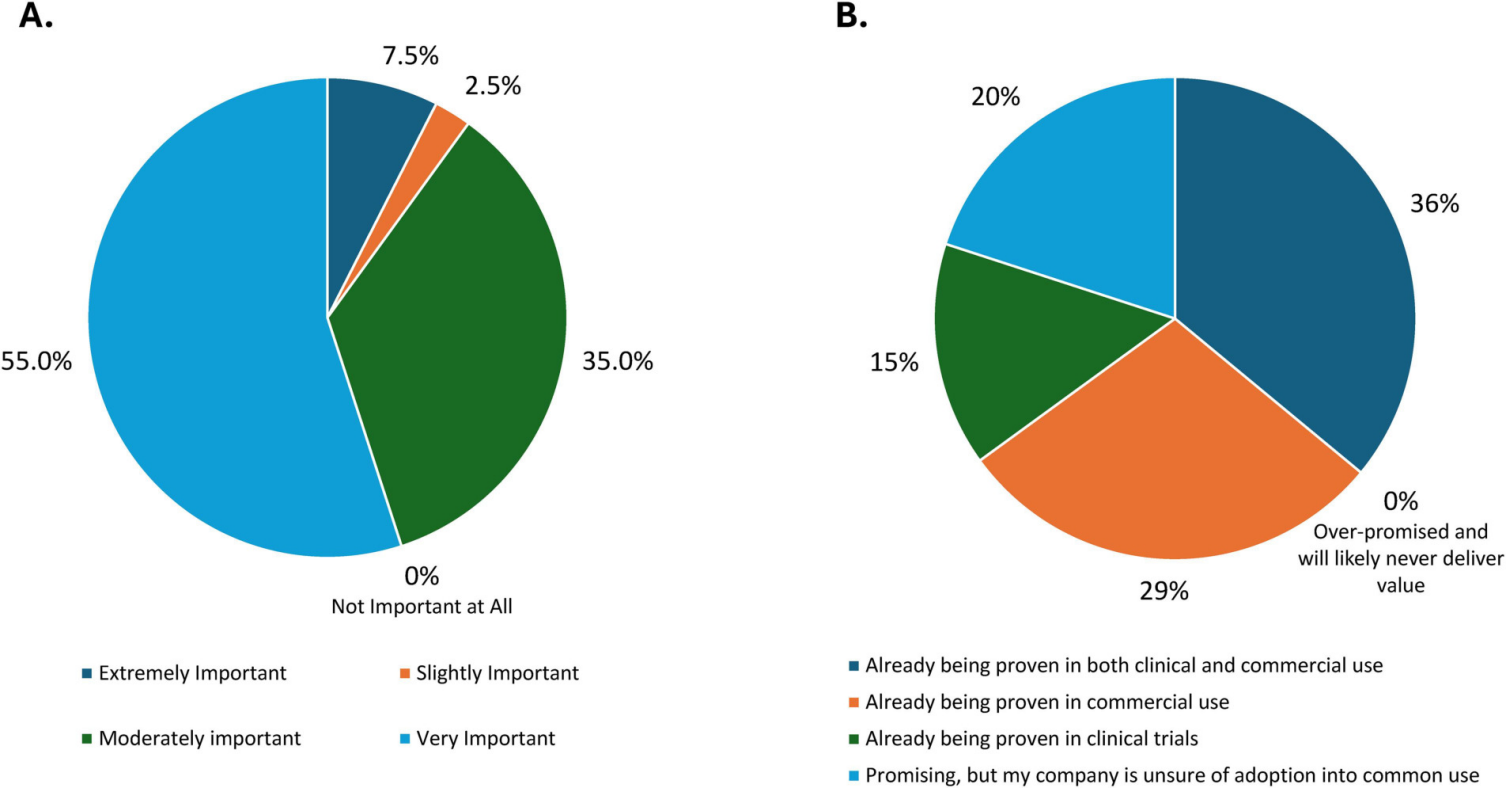
Perceived value of connected drug delivery devices in **(A)** achieving business objectives and **(B)** clinical trials and commercial settings.

Pharmaceutical, biotechnology, and digital health companies are pursuing connectivity features and investing in bringing digitally connected SC drug delivery devices to market. Nearly half (47.50%) of respondents' companies were already implementing connected SC drug delivery devices in both clinical trials and commercial settings. Among individuals who had only used connected devices in clinical trials or commercial settings but not both, there was a higher rate of using connected devices in clinical trials (15.00%) vs. commercial settings (8.75%) across company types, with the highest usage in large-cap pharmaceutical companies (Table 2). Moreover, 27.50% of participants reported that their companies were exploring concepts associated with connected drug delivery devices but had not yet used them in clinical trials or commercial settings, while only 1.25% reported that their company had not considered developing connected drug delivery devices, and no participant reported that their company had explored the development of connected drug delivery devices but was no longer interested in pursuing their development. Nearly all participants (97.5%) reported that introducing connectivity and data acquisition capabilities to their drug delivery devices was at least “moderately important” to achieving business objectives, with nearly two-thirds (63%) reporting that connectivity was “extremely” or “very important” and none reporting that connectivity was “not important at all” (Figure 2A).

**Table 2:** Relative perceived value of connectivity and data produced by connected devices in clinical trials vs. during commercial use.

Characteristic	Clinical trials (*n* = 38, 47.5%)	Commercial settings (*n* = 42, 52.5%)
Specialization	*n* (%)	*n* (%)
Combination product development	12 (31.58)	8 (19.05)
Commercial	8 (21.05)	12 (28.57)
Digital health	9 (23.68)	11 (26.19)
Medical affairs	9 (23.68)	11 (26.19)
Company type	*n* (%)	*n* (%)
Large-cap pharma	10 (26.32)	21 (50.00)
Mid-cap pharma	14 (36.84)	7 (16.67)
Small-cap pharma	8 (21.05)	5 (11.91)
Micro-cap pharma	3 (7.89)	5 (11.91)
Digital health solution developer	3 (7.89)	4 (9.52)

All data shown as *n* (%).

Most (80%) respondents reported that the value of connected SC drug delivery devices was “already being proven” in clinical trials and/or commercial use, with a small proportion (20%) reporting they were “promising,” and none reporting they were “over-promised and will likely never deliver value” (Figure 2B). Respondents were asked whether connectivity and data acquisition capabilities deliver more value in either clinical trials or commercial use; results showed mixed opinions, with an almost equal split in responses (47.5% clinical trial use, 52.5% commercial use). With respect to specialization area, 40% of combination product development, 60% of commercial, and 56% of both digital and medical affairs participants thought that there was more value in commercial settings vs. clinical trials (Table 2). Several important themes emerged from responses supporting connected SC drug delivery devices having more value in clinical trial use or commercial settings, as follows:

1. Clinical trial use
     1.1 Data quality and integrity:
    Accurate data recording without bias for patient compliance or technique.—Vice President of Medical Affairs, Mid-Cap Pharma/Biotech
    Greater data quality, speed of data collection and improved data reusability.—Vice President of Medical Affairs, Mid-Cap Pharma/Biotech
     1.2 Regulatory support:
    Clinical Trials are pivotal for the approval of any product and having any and all applicable data and anomalies for those studies for regulatory purposes can enhance the quality of your report and submission and chances of approvals.— Project/Program Management, Large-Cap Pharma/Biotech
    It's easier and quicker to provide evidence of protocol compliance to regulatory authorities.—Vice President of Medical Affairs, Small-Cap Pharma/Biotech
     1.3 Efficacy demonstration:
    Clinical trials are where outcome based data [is] most useful.—Vice President of Combination Product or Device Development, Mid-Cap Pharma/Biotech
    Control for dose and immediate endpoint reporting. — Medical Director, Small-Cap Pharma/Biotech
     1.4 Early development value:
    Helps to develop a commercially acceptable combination.—Manager/Director of Combination Product or Device Development, Large-Cap Pharma/Biotech
    Ability to adapt clinical protocol based on patient's response.—Vice President of Combination Product or Device Development, Small-Cap Pharma/Biotech
     1.5 Safety monitoring:
    Information required before risk to main population.—Engineer/Senior Engineer, Small-Cap Pharma/Biotech
    Adherence during trial. Dose finding. Adverse events. Reasons for discontinuation.—Digital Health Lead, Mid-Cap Pharma/Biotech
2. Commercial use
  2.1 Real-world evidence:
    In the commercial world, using connected tech gives us a real edge over traditional clinical trials. It's like we're getting a live feed of data on how our drugs work out there in the real world, beyond the controlled setting of a trial.—Director/Vice President of Marketing, Large-Cap Pharma/Biotech
    Real world data in a less controlled environment.—General Manager, Small-Cap Pharma/Biotech
  2.2 Patient adherence impact:
    Adherence persistence of associated drug improves scripts and revenue.—Director/Vice President of Marketing, Large-Cap Pharma/Biotech
    Because of the real-world adherence tracking to ensure that patients take their meds on time and as often as they should.—Director/Vice President of Marketing, Large-Cap Pharma/Biotech
  2.3 Market differentiation:
    *To use as a differentiation and focus on treatment experience.*—Director/Vice President of Marketing, Mid-Cap Pharma/Biotech
    *Provides a differentiator for patients and providers, improves care and adherence.*—Sales Lead, Small-Cap Pharma/Biotech)
  2.4 Healthcare cost reduction:
    It provides more functionality to the drug delivery process and saves time and money for all those. Involved, patient, care taker, provider etc.—Manager/Director of Combination Product or Device Development, Mid-Cap Pharma/Biotech
    This [leads] to better patient outcomes and cheaper overall care, which is positive for every player in the biopharma ecosystem.—Director/Vice President of Marketing, Large-Cap Pharma/Biotech
  2.5 Patient support enhancement:
    Gives more information to the patients and make them understand better their pathology.—Field Medical (Medical Science Liaison), Large-Cap Pharma/Biotech
    Scale and scope of impact + real time intervention and support.—Project/Program Management, Large-Cap Pharma/Biotech

### 3.3 Therapeutic areas of most interest for connected SC drug delivery devices

**Figure 3:**
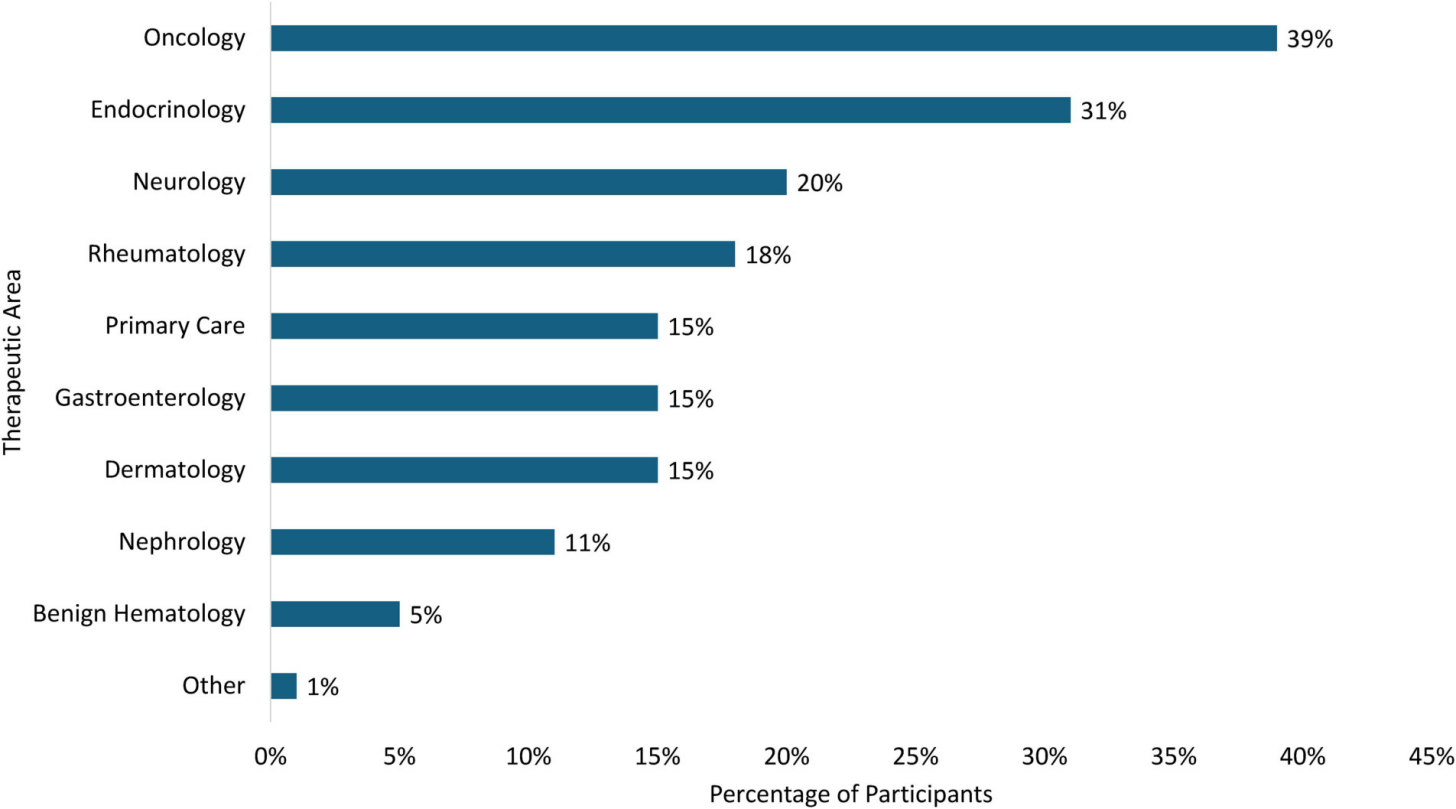
Potential therapeutic areas for connected drug delivery devices.

The most heavily considered therapeutic areas for connected SC drug delivery devices in either clinical trials or commercial settings were oncology and endocrinology. A total of 39% of respondents considered use in oncology across a wide range of disease states and 31% considered use in endocrinology, with most referencing potential use in diabetes (Figure 3).

### 3.4 Benefits of connected SC drug delivery devices for stakeholder groups

**Figure 4:**
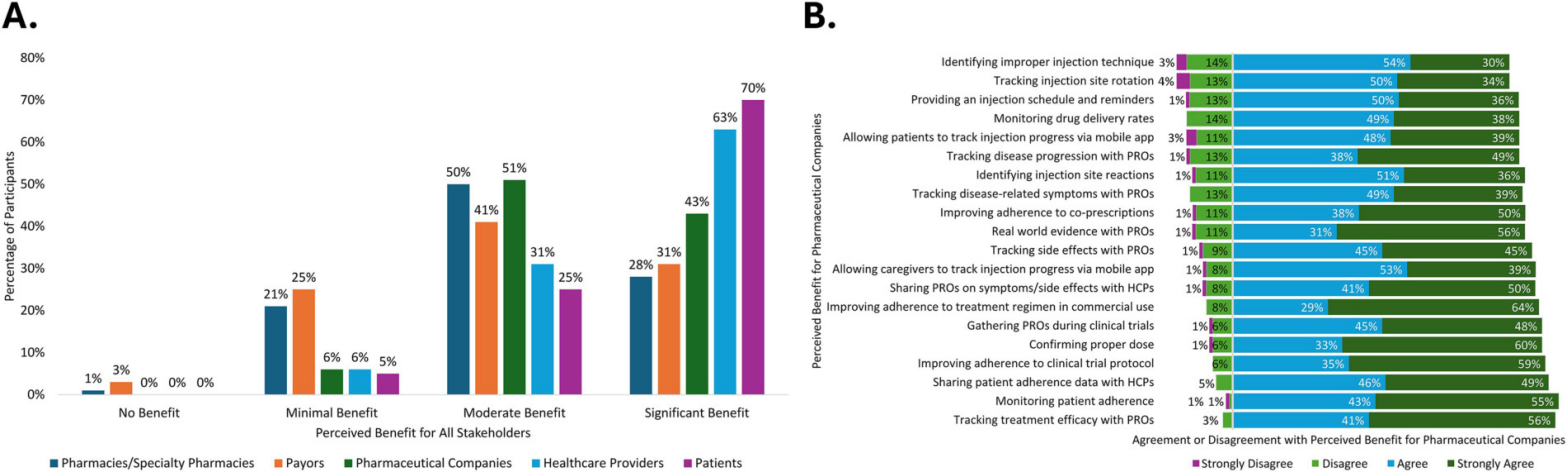
Potential benefit of connected drug delivery devices to **(A)** all stakeholders and **(B)** pharmaceutical companies.

Connected SC drug delivery devices and the data they produce offer the opportunity to provide value to all pharmaceutical stakeholders (patients, healthcare providers, payers, pharmacies, and pharmaceutical companies). Over 90% of respondents reported that all stakeholders could receive either “moderate benefit” or “significant benefit” from the connected devices, with patients and healthcare providers being the highest-rated stakeholders who could receive “significant benefit” (Figure 4A).

Respondents were provided with 20 statements describing potential benefits that connected SC drug delivery devices and associated digital tools could provide their companies. They were then asked to rate their level of agreement with each statement of potential benefits. Response options were on a scale of 1–4 (1: strongly disagree, 4: strongly agree). More than 80% of respondents “agreed” or “strongly agreed” with all statements describing the potential benefits. Four of the five highest-rated benefits were related to treatment adherence and monitoring, with “improving adherence to treatment regimen in commercial use” and “confirming the proper dose” being the highest-rated benefits to their companies (Figure 4B).

### 3.5 Measurements of return on investment in connected SC drug delivery devices

**Figure 5:**
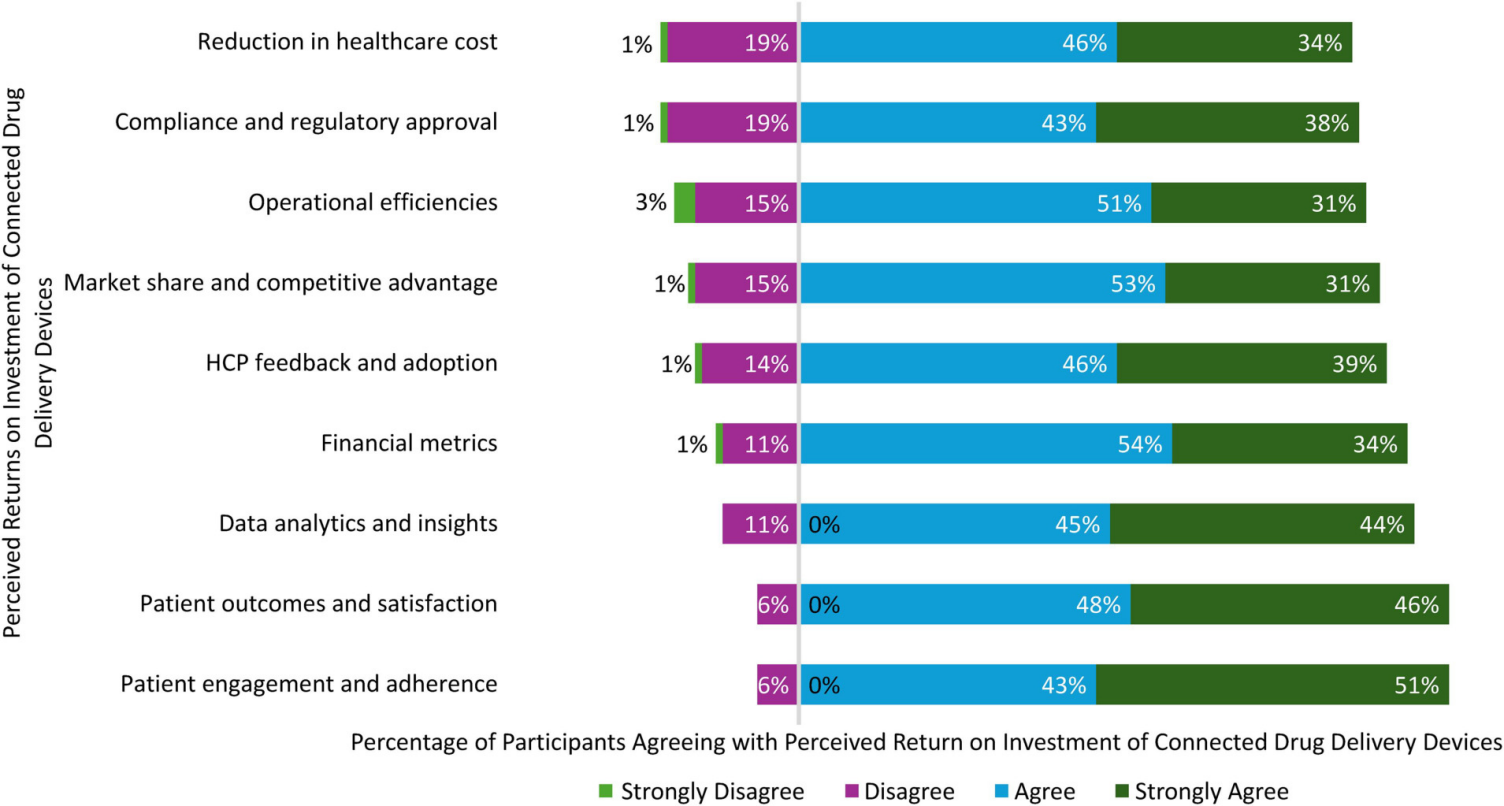
Pharmaceutical company return on investment of connected drug delivery devices.

Additionally, respondents were asked to rate nine pre-defined metrics associated with how return on investment (ROI) for connected SC drug delivery devices may be measured by their organization. Up to 80% of all respondents “agreed” or “strongly agreed” that the metrics described how their organization assesses ROI for these devices. The two highest-rated factors associated with ROI were patient focused: “patient engagement and adherence” and “patient outcomes and satisfaction,” followed by “data analytics and insights” and “financial metrics” (Figure 5).

### 3.6 Internal challenges to development and adoption

**Figure 6:**
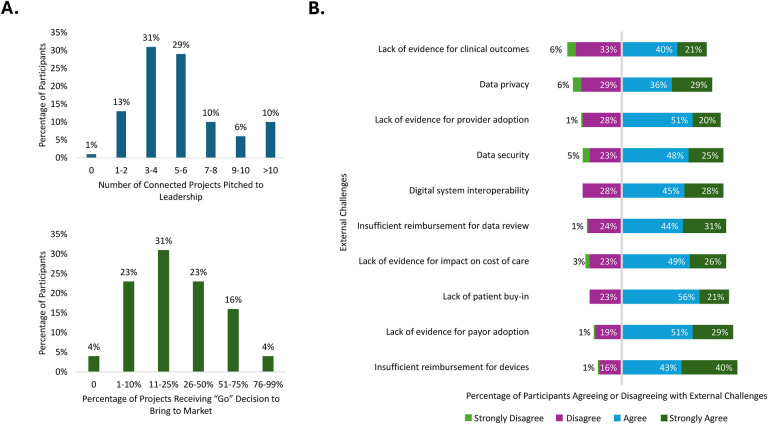
Barriers to the development and adoption of connected drug delivery devices: **(A)** internal challenges and **(B)** external challenges.

Participants understood the potential value of connected SC drug delivery devices and recognized that there are barriers to the development and adoption of these digital solutions. While respondents reported that these devices are being thoroughly considered in the pharmaceutical, biotechnology, and digital health industries, challenges remain with gaining internal endorsement for such projects, as shown by the low rate of approval by leadership to move them forward. Respondents reported a high number of development proposals to leadership, with three or more proposals reported by 86% of respondents. Of those considered by leadership, 80% of respondents estimated that less than half received internal approval and were brought to market (Figure 6A). The primary decision-makers included Vice Presidents of Combination Product or Device Development, Vice Presidents of Medical Affairs, and Medical Directors (Figure 1). Internal challenges identified by respondents were due mostly to how companies attain internal endorsement. A lack of evidence demonstrating the overall value of connected SC drug delivery devices to all stakeholders was identified as a key barrier to gaining internal endorsement.

### 3.7 Strategies for gaining internal endorsement for connected SC drug delivery devices

Respondents were asked an open-ended question about what approaches were considered useful in gaining internal endorsement on projects related to connected SC drug delivery devices. The free-text responses were coded and analyzed. The four most frequently mentioned methods (and representative responses) were as follows:

1. Developing data-driven support for the proposed value proposition and return on investment (33.75% of responses):
  Building a strong case with data is key by showcasing studies on the positive impact of medication adherence on patient outcomes and healthcare costs.—Medical Science Liaison/Medical Affairs, Mid-Cap Pharma/Biotech
  Data-driven Approach, Focus on ROI.—Manager-Director/Combination Product Development, Large-Cap Pharma/Biotech
  Review of relevant data in support of the device. Review of long-term potential benefit and cost savings.—Project Management/Combination Product Development, Large-Cap Pharma/Biotech
2. Creating formal value propositions and business models through business case development (26.3% of responses):
  ROI estimation and feasibility assessment.—Manager/Director of Combination Product or Device Development, Large-Cap Pharma/Biotech
  Ensuring: a) a very good, demonstrated ROI, b) a complexity level that can be managed or held in check, and c) excellent and consistent data collection.—Health Economic Leads (HEOR), Mid-Cap Pharma/Biotech
  Factual presentation and grounded estimate in ROI.—Manager/Director Combination Product Development, Digital Health Solution Developer
  Brought in an external consultant to produce a business case with related ROI and sales forecast to present to senior leaders.—Director/Vice President of Marketing, Large-Cap Pharma/Biotech
3. Engaging cross-functional stakeholders throughout the development process (25% of responses)
  Cross-functional collaboration early on with experts around the table to discuss pros/cons and make an informed decision.—Medical Director/Medical Affairs, Large-Cap Pharma/Biotech.
  Cross-Functional Collaboration: Involve key stakeholders from various departments early on to consider different perspectives, address challenges proactively, and gain buy-in.—Health Economics Lead/Digital Health, Digital Health Solution Provider.
  Significant cross functional engagement between development and commercialization.—Vice President of Combination Product Development, Large-Cap Pharma/Biotech.
  Cross functional team's involvement [from] the start of the project. Market research to identify the best value proposition to different stakeholders. Workshop to align with commercial teams and integrate their market feedback.—Drug Delivery Device Lead/Digital Health, Large-Cap Pharma/Biotech.
4. Executing proof-of-concept and pilot studies (16.25% of responses):
  Actual demonstrations and Proof of Concept (POC) studies.—Manager-Director/Combination Product Development, Large-Cap Pharma/Biotech
  Demonstrating the value proposition through pilot projects.—Director/Vice President of Marketing/Commercial, Micro-Cap Pharma/Biotech
  Proof of Concept: Developing a prototype or proof of concept to demonstrate the feasibility and potential impact of the device, making the benefits tangible.—General Manager/Digital Health, Micro-Cap Pharma/Biotech.

These findings suggest that pharmaceutical companies seeking to gain internal buy-in for connected SC drug delivery device projects should prioritize:

1. Building robust evidence-gathering strategies to support data-driven decision-making,
2. Developing formal business cases that demonstrate value with clear projections of return on investment,
3. Engaging diverse stakeholders early and throughout the development process, and
4. Conducting pilot studies to demonstrate feasibility and value.

The results further indicate that a multifaceted approach that combines these elements is likely to be more successful than relying on any single strategy.

### 3.8 External challenges for development and adoption

In addition to challenges within companies, there were closely related external factors and stakeholders affecting the development and adoption of connected SC drug delivery devices. Respondents were provided with 10 statements describing potential external barriers and were asked to rate their agreement with each statement on a scale of 1–4 (1: strongly disagree, 4: strongly agree). More than 60% of respondents “agreed” or “strongly agreed” with each statement regarding external barriers. The two highest-rated barriers were related to reimbursement, where ≥80% of respondents “agreed” or “strongly agreed” that there was “insufficient reimbursement for devices” and/or a “lack of evidence for payer adoption” (Figure 6B).

Respondents were asked an open-ended question regarding what they considered to be the biggest barriers to the adoption of connected SC drug delivery devices. The free-text responses were coded and analyzed. Cost was the most frequently cited external barrier to development and adoption, with 43% of respondents mentioning cost in their free-text responses. Issues with patient acceptance and education and training for proper device use were also frequently cited and identified as key barriers by 31% of respondents. Representative responses were as follows:

The biggest challenges, include, 1) reducing the complexity of the connected device for numerous stakeholders, 2) ensuring good data collection that should be provided by the connected device, and 3) ensuring that it makes financial sense from a payer perspective.—Medical Affairs, Medical Affairs, Mid-Cap Pharma/Biotech

Price point for the connected device; insurance companies are hesitant to pay for the device as it adds to their cost structure; there is added cost to pharma companies in having to provide IT support services to patients having issues with the device; regulatory approval can be a challenge as well.—Commercial, Large-Cap Pharma/Biotech

Age of patients and economic factors, Price, training of the staff.—Medical Affairs, Large-Cap, Pharma/Biotech

The adoption of connected drug delivery devices faces significant barriers, including regulatory hurdles, data security concerns, cost considerations for project development. Also, user acceptance.—Commercial, Micro-cap Pharma/Biotech

Navigating the adoption of connected drug delivery devices isn't without its hurdles. First off, there’s the privacy and security concern. With all this patient data floating around, ensuring it’s locked down tight is a massive priority, but it’s also a complex challenge. Then, we've got the cost issue. Developing these smart devices and the infrastructure to support them doesn't come cheap, and not everyone’s convinced about shelling out the extra cash without clear evidence of the return on investment.—Digital Health, Large-Cap Pharma/Biotech

## 4 Discussion

To our knowledge, this is the first study to collect pharmaceutical technology stakeholders’ perspectives on connected SC drug delivery device development. Respondents expressed recognition of the potential value of these devices while highlighting specific challenges that must be addressed for their successful implementation. Given that a clear need or gap aligned with core business objectives is required to justify investment in connected healthcare initiatives and the most compelling business cases for their development include multiple ways that these devices can impact brand revenue (33), this survey may offer insights that organizations could consider when evaluating the development and clinical implementation of connected SC devices.

For clinical trial use, perceived benefits included data quality and integrity, regulatory support, efficacy demonstration, early development value, and safety monitoring. Connected devices can be used to collect more objective data than patient-reported outcomes, which are often affected by recall and desirability bias, in order to support regulatory submissions, demonstrate drug effectiveness pre-commercialization, guide early development decisions and protocol adjustments, and help detect safety signals before broader patient exposure (33). They can also be used to accelerate recruitment; provide flexibility and optionality for participants by facilitating digital recruitment, consenting, and data collection; and their ability to improve adherence can support the powering of trials and the accuracy of the data they collect (33).

For commercial use, benefits included real-world evidence collection, patient adherence improvement, market differentiation, healthcare cost reduction, and patient support enhancement. Four of the five highest-rated benefits of connected drug delivery devices were related to treatment adherence and monitoring, with “improving adherence to treatment regimen in commercial use” and “confirming the proper dose” being the highest-rated benefits. Some studies have reported associations between connected devices and improved adherence. In multiple sclerosis, studies of the RebiSmart device showed adherence rates of 92.6–96.5% and demonstrated a correlation between adherence and reduced relapse rates (24, 27). Similarly, in growth disorders, connected devices showed improved clinical outcomes through better adherence monitoring (25). Additionally, the correlation between high adherence and efficacy outcomes reported in a review of five connected devices that complement drug treatments (e.g., RebiSmart, BETACONNECT) offers the potential to complement value-based reimbursement models (20).

This correlation between adherence and efficacy outcomes has been found across a wide range of therapeutic areas, including cardiology (34, 35), dermatology (36), endocrinology (37), infectious disease (38), neurology (39), oncology (40), psychiatry (41–43), respiratory medicine (44, 45), and rheumatology (46). While study respondents were experienced with developing SC drug delivery devices across a range of therapeutic areas, oncology and endocrinology were the two most commonly identified therapeutic areas that respondents believed could most benefit from connected SC drug delivery devices. This aligns with market trends showing increased deal-making (in-licensing and out-licensing activities) in oncology (a 142% increase from 2013 to 2017) (19). While oncology care in the US is provided mostly in clinic, in the UK it is most provided in the home. Connected healthcare might help providers feel more confident about in-home oncology care by providing oversight via connected platforms. The focus on endocrinology is also supported by successful implementations in diabetes management, where connected insulin delivery devices paired with continuous glucose monitors have shown improved glycemic control and reduced missed doses (47).

Recall, social desirability, and other cognitive and motivational biases are important limitations to consider when using self-report measures in clinical trials and during commercial use. Social desirability bias is extremely common, often unconscious, and can include both impression management, in which an individual wishes to alter how others perceive them, and self-deception, in which an individual adopts attitudes or behaviors in order to enhance their own self-perception (48). Baseline counseling, the informed consent process, contact with healthcare professionals, or the experience of participating in a study about a particular behavior may also alter patients’ perceptions of their behaviors and change how they define or calculate the frequency of the behavior (49). Collecting treatment, outcome, and adherence data using connected devices or mobile applications, which are not subject to these or other biases, may improve the reliability and validity of collected data compared with self-report measures. Frequent remote, digital data collection, sometimes called ecologic momentary assessment, can also eliminate the aggregation of behaviors common in self-report data and decrease the recall bias common when too long of a follow-up period is employed (49–51).

Internal challenges identified by respondents were due mostly to how companies attain internal endorsement, with the lack of evidence demonstrating the value of connected SC drug delivery devices to all stakeholders identified as a key barrier to gaining internal endorsement. However, the emphasis on cross-functional collaboration and evidence generation as key strategies for gaining internal support reflects lessons learned from successful implementations. For example, Vicore's approach to digital therapeutic development in idiopathic pulmonary fibrosis demonstrated how early stakeholder engagement and robust evidence generation can facilitate organizational buy-in (52). In partnership with Alex Therapeutics, Vicore Pharma introduced a digital cognitive behavioral therapy (CBT) tool designed to address the psychological challenges faced by patients with idiopathic pulmonary fibrosis (53). Alex Therapeutics conducted a literature review and interviews to understand disease and treatment needs and how a digital product would support clinical care, customized an appropriate platform, and conducted a regulatory analysis, while Vicore developed a clinical trial protocol (53). In the pivotal randomized controlled trial COMPANION, patients treated with the CBT tool demonstrated clinically meaningful and statistically significant improvements in anxiety symptoms compared with controls (54). This innovative use of a digital CBT tool showcased its value in addressing a critical unmet need—patients with pulmonary fibrosis report needing more information and education; support with the management of anxiety, anger, sadness, and fear; and access to peer and psychological support (55)—while illustrating a successful cross-functional partnership.

With respect to external barriers to the adoption of connected drug delivery devices, the success of digital health technologies such as connected drug delivery devices relies on patient trust in the safety and security of these technologies (56), and data security emerged as a key concern among respondents. Many devices, such as glucose measurement devices or insulin pumps, use Bluetooth or other wireless interface technologies to connect to each other and to gateways or transfer data to apps, and data and electronic health record storage and access and artificial intelligence for data interpretation are facilitated by cloud computing platforms (57). These and other aspects of connected device use pose cybersecurity risks that must be addressed appropriately by device manufacturers and system developers (57). Increasing regulatory scrutiny of digital health technologies (58), such as the implementation of General Data Protection Regulation in Europe and similar regulations globally, has also created new compliance requirements that must be addressed during development (20). However, some commercial examples like the BETACONNECT system suggest that certain challenges can be addressed in practice, although further evidence is needed.

Moreover, our findings regarding the importance of patient acceptance and education—identified by participants as an important external barrier to the adoption of connected drug delivery devices—echo conclusions from studies of connected devices and mobile health technologies. Previous examples suggest the need for comprehensive patient support programs and a clear demonstration of user benefits (28). In a cross-sectional observational study of patients with type 2 diabetes and their physicians, education about links between physical activity and eating and blood glucose, in addition to cost and medication reminders, was the factor most predictive of patient participation in a connected ecosystem program (59). The benefits of a smartphone-enabled insertable cardiac monitor for patients with hypertrophic cardiomyopathy included improved symptom clarity and reassurance due to receiving feedback from transmissions from the insertable cardiac monitor (60). Similarly, an observational study of a medical app for patients receiving trauma and orthopedic surgical care found that the most important app functions for patients were information about medication, behavioral guidelines, and medical record archival (61). Although research on connected drug delivery devices is more limited, these studies underscore the importance of patient education and the clear demonstration of user benefits in encouraging patient acceptance.

Participants suggested that approaches such as building robust evidence-gathering strategies to support data-driven decision-making, developing formal business cases that demonstrate value with clear projections of return on investment, engaging diverse stakeholders early and throughout the development process, and conducting pilot studies to demonstrate feasibility and value may help to address these challenges. As one participant wrote, “To gain alignment and internal buy-in on projects related to connected drug delivery devices, organizations often engage key stakeholders early, communicate the project's vision clearly, and address concerns proactively. Demonstrating the value proposition through pilot projects, collaborative decision-making, and providing training and education can further foster support and commitment to the initiative.” Building a strong business case bolstered by well-designed cost-effectiveness studies should support these efforts (62).

This study has several limitations. As the first survey-based assessment of internal stakeholders’ perspectives on SC drug delivery devices, it was designed to capture opinions rather than establish definitive conclusions and provides descriptive rather than inferential analyses, and the results presented here should therefore be considered hypothesis-generating rather than definitive. All members of the study team and all respondents were affiliated with pharmaceutical, device, and/or digital health organizations, some of which are involved in the development of connected drug delivery technologies. Although conflicts of interest were fully disclosed and methodological safeguards were used, these affiliations may have influenced study design, topic prioritization, and/or the interpretation of findings. Because participants were recruited based on their experience working directly with connected or digitally enabled drug delivery systems, respondents may have been more positively predisposed toward the value of connectivity than the broader population of healthcare stakeholders and may therefore have emphasized benefits and underrepresented concerns. The perspectives presented here reflect those of experts actively engaged in developing or evaluating connected SC drug delivery devices and should therefore not be interpreted as representing the full range of views across clinicians, payers, patients, or external technology developers. The survey design limits the ability to determine whether respondents’ opinions were evidence-based or derived from personal experience, and the study is subject to typical self-report limitations, including potential user error and sampling bias. Although key internal decision-makers were included, other relevant stakeholders such as clinical development, R&D, regulatory affairs, and board leadership were not, which may further narrow the diversity of viewpoints. Additionally, because most respondents were based in the United States, the generalizability of the findings to other settings is limited. Despite these limitations, the breadth and depth of respondent experience provide valuable insight into current industry sentiment regarding connected SC drug delivery technologies and the challenges and opportunities perceived by those closely involved in their development.

## 5 Conclusion

This survey study is the first to assess digitally connected SC drug delivery devices from the perspective of internal pharmaceutical stakeholders. The survey included questions about issues that are often the subject of speculation in the healthcare and digital health communities but for which there is no publicly available evidence. The opinions provided here can therefore be used to inform executive leadership about connected SC devices. Respondents reported perceiving potential value in connected SC drug delivery devices and associated companion mobile applications, particularly for acquiring real-world data, improving patient engagement and adherence, and enhancing ease-of-use for patients. The potential to improve patient adherence may be particularly important given the correlation between adherence and treatment efficacy. Indications in oncology and endocrinology were considered the most likely to benefit from connected SC drug delivery devices. The perspectives of internal pharmaceutical stakeholders on connectivity may contribute to a better understanding of current industry sentiment regarding connected healthcare.

## Data Availability

The raw data supporting the conclusions of this article will be made available by the authors, without undue reservation.
