# Supplementary material for: Value, development challenges, and strategies for gaining internal endorsement of digitally connected subcutaneous drug delivery devices: a survey of pharmaceutical stakeholders
Source: Front Digit Health. 2026 Mar 26;8:1684685. doi: 10.3389/fdgth.2026.1684685 (PMC13062299; doi:10.3389/fdgth.2026.1684685)
Supplement: Supplementary file 1 [file Datasheet1.docx]

**Connected Drug Delivery Survey**

Screening Questions

1. In which region do you currently work/reside?
2. US
3. Canada
4. Italy
5. Spain
6. UK
7. Other; please specify

2. Which of the following best describes your organization?

1. Large-cap pharma/biotech (>$10 billion market capitalization)
2. Mid-cap pharma/biotech ($2-$10 billion market capitalization)
3. Small-cap pharma/biotech ($250 million - $2 billion market capitalization)
4. Micro-cap pharma/biotech (<$250 million market capitalization)
5. An injection system manufacturer/provider
6. A digital health solution developer/provider
7. A design and engineering consultancy
8. Other; please specify

3. What best describes your primary area of specialization?

1. Medical affairs
2. Commercial
3. Combination product development
4. Digital health
5. CMC
6. Other; please specify

4. How many years of relevant experience do you have in the above specialization?

1. Less than 2 years
2. 2-3 years
3. 4-5 years
4. 6-7 year
5. 8-9 years
6. 10-11 years
7. 12-13 years
8. 14-15 years
9. More than 15 years

5. Which of the following best describes your title/role?

1. Medical Director
2. VP of Medical Affairs
3. Medical Science Liaison
4. Field Medical
5. Health Economic Lead (HEOR)
6. Brand/Asset Strategy Lead
7. Director/VP of Marketing
8. Sales Lead
9. General Manager
10. Project/Program Manager
11. Manager/Director of Combination Product or Device Development
12. VP of Combination Product or Device Development
13. Engineer/Senior Engineer
14. Drug Delivery Device Lead
15. Digital Health Lead
16. Other; please specify

6. How many subcutaneous drug-device combination products (e.g., drug + delivery device such as an autoinjector, syringe, on-body delivery system or other device) have you worked on during your career?

1. 0
2. 1-2
3. 3-4
4. 4-5
5. 5-6
6. 7-8
7. 9-10
8. More than 10

7. What is your level of experience contributing to projects that have considered the

use of subcutaneous drug delivery devices with connectivity functionality? Please rate

your level of experience on a scale of 1 to 5, where 1 is no experience and 5 is very

experienced. Note: Connectivity can be interpreted as acquiring information about device

use or injections from the delivery device, for example – an autoinjector that tracks the

date and time that injections are performed and transmits this information to a patient

companion app on a mobile device.

1. 1- No experience
2. 2
3. 3 – Some experience
4. 4
5. 5 – Experienced

8. What percentage of these subcutaneous drug-device combination products have considered the use of a connected drug delivery device?

1. 0%
2. 1-5%
3. 6-10%
4. 11-15%
5. 16-20%
6. 21-25%
7. 26-30%
8. 31-35%
9. 36-40%
10. 41-50%
11. 51-55%
12. 56-60%
13. 61-65%
14. 66-70%
15. 71-75%
16. 76-80%
17. 81-85%
18. 86-90%
19. 91-95%
20. 95-99%
21. 100%

9. What percentage of these subcutaneous combination products have considered the

use of a patient companion mobile application?

1. 0%
2. 1-5%
3. 6-10%
4. 11-15%
5. 16-20%
6. 21-25%
7. 26-30%
8. 31-35%
9. 36-40%
10. 41-50%
11. 51-55%
12. 56-60%
13. 61-65%
14. 66-70%
15. 71-75%
16. 76-80%
17. 81-85%
18. 86-90%
19. 91-95%
20. 95-99%
21. 100%

10. How involved are you in decisions around subcutaneous combination product

development?

1. I am the decision-maker
2. I am involved in the decision-making process
3. I am not involved in the decision-making process
4. I am not sure

11. What are the main therapeutic areas of your experience with subcutaneous drug

products? Please select all that apply.

1. Oncology
2. Neurology
3. Nephrology
4. Dermatology
5. Gastroenterology
6. Rheumatology
7. Endocrinology (e.g., diabetes)
8. Benign hematology
9. Primary care (e.g., obesity)
10. Other; please specify
11. None of the above

Questionnaire Questions

1. Which of the following statements best describes your company’s experience with bringing connected drug delivery devices to market?
2. We have used connected drug delivery devices in both commercial use and clinical trials
3. We have used connected drug delivery devices in commercial use only
4. We have used connected drug delivery devices in clinical trials only
5. We are exploring concepts associated with connected drug delivery devices, but have not utilized connected devices in clinical trials or commercial use thus far
6. We have previously explored the development of connected drug delivery devices, but are no longer interested in pursuing the development of such offerings
7. We have not considered developing connected drug delivery devices
8. Please detail why your organization has decided to no longer pursue the development of connected drug delivery devices.
9. Which of the following clinical specialties have you considered utilizing connected drug delivery devices in either clinical trials or commercial use? Please select all specialties that apply and indicate the relevant disease states.
10. Oncology; please specify
11. Neurology; please specify
12. Nephrology; please specify
13. Dermatology; please specify
14. Gastroenterology; please specify
15. Rheumatology; please specify
16. Endocrinology (e.g., diabetes); please specify
17. Benign hematology; please specify
18. Primary care (e.g., obesity); please specify
19. Other; please specify; please specify
20. None of the above
21. Please assess each factor listed below on its relative importance when it comes to identifying appropriate patient populations for the use of connected drug delivery devices. Please rate the following on a scale of 1 to 4, where 1 is not at all important at all and 4 is very important.
22. Patient population age distribution
23. Patient population technology aptitude
24. Patient population socioeconomic factors
25. Known/historical adherence challenges in the patient population
26. Treatment indication
27. Therapy regimen complexity
28. Other; please specify
29. What is the percent breakdown of the delivery device format that has been utilized to administer subcutaneous combination products in your past projects? Table must sum to 100%.
30. Autoinjector
    1. 0
    2. 10
    3. 20
    4. 25
    5. 30
    6. 33
    7. 35
    8. 40
    9. 45
    10. 50
    11. 60
    12. 70
    13. 80
    14. 90
    15. 100
31. Syringe
    1. 0
    2. 10
    3. 20
    4. 25
    5. 30
    6. 33
    7. 35
    8. 40
    9. 45
    10. 50
    11. 60
    12. 70
    13. 80
    14. 90
    15. 100
32. On-body delivery system
    1. 0
    2. 10
    3. 20
    4. 25
    5. 30
    6. 33
    7. 35
    8. 40
    9. 45
    10. 50
    11. 60
    12. 70
    13. 80
    14. 90
    15. 100
33. Other; please specify
    1. 0
    2. 10
    3. 20
    4. 25
    5. 30
    6. 33
    7. 35
    8. 40
    9. 45
    10. 50
    11. 60
    12. 70
    13. 80
    14. 90
    15. 100
34. In your opinion, what is the most promising value proposition associated with connected drug delivery devices? Please describe the benefits provided, and to whom (considering all stakeholders - patients, healthcare providers, pharmaceutical companies, payors etc).
35. The following table presents several statements describing potential benefits of connected drug delivery devices and associated digital tools (e.g., patient companion applications). Please review each statement, then rate your level of agreement that such a benefit would provide value to your organization. Please rate the following on a scale of 1 to 4, where 1 is strongly disagree and 4 is strongly agree.
36. Monitoring patient adherence to their treatment regimen would be of value to my organization
37. Providing an injection schedule and reminders to patients would be of value to my organization
38. Gathering real-world evidence with patient reported outcomes would be of value to my organization
39. Tracking disease related symptoms with patient reported outcomes would be of value to my organization
40. Tracking side effects with patient reported outcomes would be of value to my organization
41. Tracking disease progression with patient reported outcomes would be of value to my organization
42. Tracking treatment efficacy with patient reported outcomes would be of value to my organization
43. Identifying improper injection device technique/usage would be of value to my organization
44. Tracking injection site/location to ensure proper site rotation would be of value to my organization
45. Confirming that the proper dose/volume has been delivered would be of value to my organization
46. Monitoring the injection/drug delivery rates would be of value to my organization
47. Allowing patients to track injection progress (e.g., injection rate and estimated time to completion) via a mobile application would be of value to my organization
48. Allowing caregivers to track injection progress (e.g., injection rate and estimated time to completion) via a mobile application would be of value to my organization
49. Identifying injection site reactions would be of value to my organization
50. Improving patient adherence to treatment protocol/regimen in clinical trials would be of value to my organization
51. Improving patient adherence to treatment protocol in commercial use would be of value to my organization
52. Gathering patient reported outcomes during clinical trials would be of value to my organization
53. Improving patient adherence to medications co-prescribed with the primary therapy would be of value to my organization
54. Sharing patient therapy adherence data with healthcare providers would be of value to my organization
55. Sharing patient reported symptom/side effect tracking data with healthcare providers would be of value to my organization
56. Other; please specify
57. Considering the value of connected delivery devices, and the data they produce, rate the benefits such solutions may provide each of the stakeholders in the list below. Please rate the benefit for each stakeholder on a scale of 1 to 4, where 1 is no benefit and 4 is significant benefit.
58. Patients
59. Pharmaceutical companies
60. Healthcare providers
61. Pharmacies/specialty pharmacies
62. Payors
63. In your opinion, does connectivity and the data produced by such devices provide more value in clinical trials, or during commercial use?
64. Clinical trials
65. Commercial use
66. Why do you feel connectivity provides more value in your answer to Q9?
67. Which of the following statements best aligns with your perception of the value that connected drug delivery devices may deliver?
68. The value of connected drug delivery devices is already being proven in both clinical and commercial use
69. The value of connected drug delivery devices is already being proven in commercial use
70. The value of connected drug delivery devices is already being proven in clinical trials
71. The value of connected drug delivery devices is promising, but my company is unsure that it will be adopted into common use
72. The value of connected drug delivery devices is over-promised and will likely never deliver value
73. What are the biggest barriers to the adoption of connected drug delivery devices?
74. The table below contains several statements describing potential barriers that may hinder the adoption of connected drug delivery devices by external stakeholders (those outside of your organization). Please indicate your level of agreement with each statement. Please rate each statement on a scale of 1 to 4 where 1 is strongly disagree and 4 is strongly agree.
75. Concerns about data privacy present a barrier to the adoption of connected drug delivery devices
76. Concerns about data security present a barrier to the adoption of connected drug delivery devices
77. A lack of clear evidence demonstrating effectiveness of connected drug delivery devices presents a barrier to healthcare providers adopting connected drug delivery devices
78. A lack of clear evidence demonstrating effectiveness of connected drug delivery devices presents a barrier to payors adopting connected drug delivery devices
79. A lack of evidence demonstrating an impact on clinical outcomes presents a barrier to the adoption of connected drug delivery devices
80. A lack of evidence demonstrating an impact on the cost of care presents a barrier to the adoption of connected drug delivery devices
81. Insufficient reimbursement for connected drug delivery devices presents barriers to the adoption of connected drug delivery devices
82. Insufficient reimbursement for the time required of healthcare providers to review data produced by connected drug delivery devices presents a barrier to the adoption of connected drug delivery devices
83. Limited interoperability with existing information technology (e.g., integrating data into EMR) presents a barrier to the adoption of connected drug delivery devices
84. A lack of patient buy-in and engagement with such solutions present a barrier to the adoption of connected drug delivery devices
85. How critical is introducing connectivity and data acquisition capabilities to your drug delivery devices to achieving important business objectives? Please rate on a scale of 1 to 5, where 1 is not at all important and 5 is extremely important.
86. 1 – Not at all important
87. 2 – Slightly important
88. 3 – Moderately important
89. 4 – Very important
90. 5 – Extremely important
91. The table below contains several statements describing ways that return on investment for connected drug delivery device initiatives may be measured. Please indicate your level of agreement with each statement considering how your organization assesses return on investment. Please rate each statement on a scale of 1 to 4 where 1 is strongly disagree and 4 is strongly agree.
92. Financial Metrics: We evaluate ROI by measuring financial impact such as net profit, cost savings, and revenue growth
93. Patient Outcomes and Satisfaction: We evaluate ROI by measuring improvements in patient outcomes and satisfaction levels
94. Operational Efficiency: We evaluate ROI by measuring improvements in operational efficiency, such as reduced administration time, fewer errors, and streamlined workflows
95. Market Share and Competitive Advantage: We evaluate ROI by measuring gains in market share, enhanced competitive positioning, and the ability to enter new markets
96. Compliance and Regulatory Approvals: We evaluate ROI by achieving regulatory approvals and compliance with healthcare regulations
97. Data Analytics and Insights: We evaluate ROI by measuring the value of data analytics and insights gained from device usage, which can inform future product development and personalized medicine approaches
98. Healthcare Professional (HCP) Feedback and Adoption: We evaluate ROI by measuring the feedback received from healthcare professionals regarding ease of use, the impact on treatment protocols, and the rate of adoption within clinical settings
99. Reduction in Healthcare Costs: We evaluate ROI by measuring the overall healthcare cost reduction for patients and insurers, including fewer hospital visits and decreased need for other healthcare services
100. Patient Engagement and Adherence: We evaluate ROI by measuring improvements in patient engagement and medication adherence rates, recognizing these factors' impact on treatment success and long-term outcomes
101. Technology Integration and Ecosystem Expansion: We evaluate ROI by measuring the success of integrating the device into existing healthcare IT ecosystems and the creation of new partnerships or ecosystems
102. Scalability and Flexibility of the Solution: We evaluate ROI by measuring the scalability of the technology and its flexibility to adapt to different drugs or therapeutic areas
103. Brand Image and Patient Trust: We evaluate ROI by measuring improvements in brand image and trust from patients and healthcare providers, acknowledging the intangible benefits of digital health initiatives
104. Regulatory Compliance and Risk Management: We evaluate ROI by measuring how connectivity and associated data has enhanced regulatory compliance, risk management, and the ability to navigate post-market surveillance requirements effectively
105. Other; please specify
106. Who (what departments and stakeholders) is involved in projects related to connected drug delivery devices? Please select all that apply.
107. Medical Director
108. VP of Medical Affairs
109. Medical Science Liaison
110. Field Medical
111. Health Economics Lead (HEOR)
112. Brand/Asset Strategy Lead
113. Director/VP of Marketing
114. Sales Lead
115. General Manager
116. Project/Program Manager
117. Manager/Director of Combination Product or Device Development
118. VP of Combination Product or Device Development
119. Engineer/Senior Engineer
120. Drug Delivery Device Lead
121. Digital Health Lead
122. Other; please specify
123. Who (what departments and stakeholders) are the primary decision makers when determining whether to implement features such as connectivity into drug delivery devices developed/offered by your company? Please select all that apply.
124. Medical Director
125. VP of Medical Affairs
126. Medical Science Liaison
127. Field Medical
128. Health Economics Lead (HEOR)
129. Brand/Asset Strategy Lead
130. Director/VP of Marketing
131. Sales Lead
132. General Manager
133. Project/Program Manager
134. Manager/Director of Combination Product or Device Development
135. VP of Combination Product or Device Development
136. Engineer/Senior Engineer
137. Drug Delivery Device Lead
138. Digital Health Lead
139. Other; please specify
140. Which of the following statements best aligns with your organization’s approach to developing and managing the digital infrastructure needed to store and analyze data produced by connected drug delivery devices and associated mobile applications?
141. Developed internally
142. Mix of internally developed and outsourced
143. All externally developed and managed
144. I am not sure
145. What level of involvement have outside vendors played in presenting connected drug delivery initiates to internal decision makers?
146. Not involved
147. Slightly involved
148. Moderately involved
149. Significantly involved
150. Led only the communication
151. Exclusively responsible
152. Not sure
153. In your best estimation, how many projects related to introducing connectivity to drug delivery devices have been presented, or “pitched” to leadership within your organization?
154. 0
155. 1-2
156. 3-4
157. 5-6
158. 7-8
159. 9-10
160. >10
161. In your best estimation, what percentage of the projects considering introducing connectivity to drug delivery devices have received a “go” decision from leadership - meaning they are approved and funded to bring to market?
162. 0%
163. 1-10%
164. 11-25%
165. 26-50%
166. 51-75%
167. 76-99%
168. 100%
169. What level of evidence/study is typically executed prior to launch of a connected drug delivery device? Please select all that apply.
170. Formative user research
171. Usability studies
172. Clinical trials
173. No connected feature-specific studies are typically executed
174. Other; please specify
175. Not sure
176. You indicated that clinical trials would be necessary prior to launch of a connected drug delivery device, please specify what phase trials (I, II, II).
177. The table below contains several statements describing potential challenges to gaining internal buy in on connected drug delivery devices projects within your organization. Please indicate your opinion on how much of a challenge each item can be. Please rate each statement on a scale of 1 to 4, where 1 is not a challenge and 4 is significant challenge.
178. Lack of evidence to support value to external stakeholders
179. Lack of evidence to support value to your organization
180. Regulatory uncertainty
181. Lack of internal expertise in digital solutions
182. Lack of digital infrastructure to house and manage data properly
183. The multidisciplinary nature of the development for such offerings
184. Introducing these types of capabilities extends the timeline to bring the drug product to market
185. Legal risk associated with data management
186. High cost of implementing such solutions
187. Lack of a return on investment for your organization
188. Resistance to change within the organization
189. Technical limitations (e.g., sensor and data communication technology capabilities and physical size)
190. Other; please specify
191. What approaches or methods have you used, or seen used to gain alignment and internal buy-in on projects related to connected drug delivery devices?
